# Pancreatic Ductal Adenocarcinoma: Current and Evolving Therapies

**DOI:** 10.3390/ijms18071338

**Published:** 2017-06-22

**Authors:** Aleksandra Adamska, Alice Domenichini, Marco Falasca

**Affiliations:** Metabolic Signalling Group, School of Biomedical Sciences, Curtin Health Innovation Research Institute, Curtin University, Perth, WA 6102, Australia; aadamskaa77@gmail.com (A.A.); alice.domenchini@curtin.edu.au (A.D.)

**Keywords:** PDAC, chemotherapy, gemcitabine, Abraxane, FOLFIRINOX, combination therapies, targeted therapies

## Abstract

Pancreatic ductal adenocarcinoma (PDAC), which constitutes 90% of pancreatic cancers, is the fourth leading cause of cancer-related deaths in the world. Due to the broad heterogeneity of genetic mutations and dense stromal environment, PDAC belongs to one of the most chemoresistant cancers. Most of the available treatments are palliative, with the objective of relieving disease-related symptoms and prolonging survival. Currently, available therapeutic options are surgery, radiation, chemotherapy, immunotherapy, and use of targeted drugs. However, thus far, therapies targeting cancer-associated molecular pathways have not given satisfactory results; this is due in part to the rapid upregulation of compensatory alternative pathways as well as dense desmoplastic reaction. In this review, we summarize currently available therapies and clinical trials, directed towards a plethora of pathways and components dysregulated during PDAC carcinogenesis. Emerging trends towards targeted therapies as the most promising approach will also be discussed.

## 1. Introduction

Pancreatic ductal adenocarcinoma (PDAC) is one of the most aggressive solid malignancies. Despite quite a low incidence, it remains the fourth leading cause of cancer-related deaths in the modern world, mainly because of dismal diagnosis [1]. In the last decades, significant improvements have been achieved in the screening and therapy of different solid cancers, highly incrementing patients’ chance for cure. Nevertheless, despite the advancement in pancreatic cancer research, the mortality to incidence ratio has not experienced significant revision over the last few decades. The five-year survival rate remains just around 5–7% and one-year survival is achieved in less than 20% of cases [2]. This grim prognosis is mainly caused by the lack of visible and distinctive symptoms and reliable biomarkers for early diagnosis as well as aggressive metastatic spread leading to poor response to treatments [3]. In fact, around 50% of diagnosed patients present with metastatic disease. Furthermore, tumour heterogeneity and plasticity cause PDAC to develop chemoresistance. Progression of the disease through consecutive stages is accompanied by accumulating morphological and genetic alterations. Consequently, aberrations in signalling pathways are observed in PDAC progression. Over-activation of many signalling pathways involved in growth and proliferation, as well as altered expression of tumour suppressor genes are regularly detected in PDAC, influencing cell proliferation, survival and invasion. The broad repertoire of genetic and metabolic remodelling allows PDAC to survive under harsh conditions and increases proliferative ability. Furthermore, recent analysis of gene expression and activity allowed for classification of observed mutations into four distinct phenotypic subtypes defined as squamous, pancreatic progenitor, immunogenic and aberrantly differentiated endocrine exocrine (ADEX) [4]. Each of the subtypes is characterized by different mutational landscape, tumour histopathological features and correlates with different prognosis. Classification of diagnosed patients into one of these four subtypes may provide substantial prognostic value and be of great therapeutic relevance, allowing for more personalized treatments. Additionally, a dense, diffuse stroma called desmoplasia, is formed around the tumour, contributing to its resistance and influencing tumour progression and invasion [5,6,7]. All described events make pancreatic cancer resistant to currently applied therapies, demanding for novel, broader approaches to improve PDAC patients’ perspectives. Conventional cytotoxic treatments, such as chemotherapy and radiotherapy, have been rather unsuccessful in improving patients’ chances for survival, offering marginal benefits. Single agent gemcitabine, as well as its combinations, failed to provide expected results, prolonging life expectancy only moderately. Similarly, disappointing effects were achieved with multidrug regimens (e.g., folinic acid-fluorouracil-irinotecan-oxaliplatin also known as FOLFIRINOX) and targeted therapies. Therefore, there is a pivotal need for development of novel, effective strategies aiming to advance current therapeutic possibilities. Improvement in the field of targeted, more personalized therapies is of high importance. Multiple preclinical and clinical studies are being developed in order to address these points; however, because most of them are in early phases, it is still too soon to draw any conclusion. In this review, we provide a broad description of the development of PDAC therapy, and introduce currently available therapies and strategies that are presently being undertaken to improve PDAC patients’ perspectives.

## 2. Disease Staging-Essential Factor in Pancreatic Ductal Adenocarcinoma (PDAC) Therapy

Treatment options for pancreatic ductal adenocarcinoma are rather limited and highly depend on the disease’s stage. Therefore, proper diagnosis and accurate staging allow for better prognosis and highly influence treatment choice and patients’ chance of survival. Multi-detector computed tomography (CT) scan accompanied by three-dimensional (3D) reconstruction is currently the first choice imaging option for preoperative staging of PDAC [8,9]. However, due to poor sensitivity in distinguishing between peritoneal and small hepatic metastasis, CT scan is not suitable to accurately predict resectability [10]. Endoscopic ultrasound, magnetic resonance imaging and laparoscopy are also used to properly classify diagnosed tumours and, with the latter, exclude intraperitoneal metastases [11,12]. Other than imaging techniques, CA19-9 levels evaluation, despite its limitations, is recommended to correctly stage PDAC, once diagnosed, as well as to assess the response to therapy [13]. From a surgical point of view, PDAC is classified based on the tumour node metastasis (TNM) system, in which primary tumour size (TX, T0–T4), regional lymph nodes (NX and N0–N1) and distant metastasis (M0–M1) are assessed [14,15]. Based on the combination of assessed TNM values, diagnosed tumours are staged due to anatomic state and divided into different prognostic groups (0–II resectable; III locally advanced, unresectable; IV metastatic unresectable). For clinical management, PDAC is divided into 4 main categories depending on the tumour extension: resectable, borderline resectable, which exhibit venous involvement of superior mesenteric vein/portal-vein (SMV/PV) and gastroduodenal artery encasement, locally advanced and metastatic. Currently, surgical resection of the pancreas with microscopically free margins remains the only realistic and potentially curative option for pancreatic cancer patients, however it is restricted to earlier disease stages. Unfortunately, at the time of diagnosis, less than 20% of patients have a resectable tumour [16]. The remaining patients frequently present tumours and metastases, which are already too widespread to be surgically removed. At this stage of the disease retroperitoneal and perineural infiltration, haematogenic dissemination and angioinvasion are observed. In particular, cancers of the body and tail of the pancreas are often detected at the late stage and they usually present major vessels involvement, such as hepatic artery or celiac axis [17]. Therefore, even despite the lack of metastasis, they are usually classified as unresectable.

## 3. Therapy for Metastatic Cancer

Once metastasized, pancreatic cancer prognosis is poor. Chemotherapy treatment remains the main option for patients with advanced and metastatic tumours. Radiation, in combination with chemotherapy, is another option for unresectable, metastatic cancer [18]. Nonetheless, the effects achieved by both approaches are mainly a mildly increased survival rate and lowered cancer-related symptoms. Moreover, due to elevated toxicity, combination chemotherapy, which is associated with slightly better outcomes, is limited only to patients with a good performance status (PS). Therefore, depending on the PS, PDAC patients may be subjected to combination or single-agent treatment. Multidrug regimens would potentially increase the patient anti-tumour response. However, they are associated with higher toxicity and greater incidence of adverse effects [19]. Nevertheless, in all therapeutic regimens, some general side effects are expected, including complications associated with a reduction in blood cell counts, vomiting and nausea, diarrhoea, constipation, mouth ulcers, poor appetite, hair loss, nervous system changes, and infertility. It has been considered that some of these adverse effects, especially blood clotting and weight loss, may be one of the reasons for the ineffectiveness of current therapies, forcing their early termination. Therefore, learning how to manage these adverse symptoms could significantly improve patients’ outcomes. Current clinical trials and available therapies are listed in Table 1 and Table 2.

### 3.1. Gemcitabine

In the earliest decades of pancreatic cancer treatment, despite considerable toxicity, 5-fluorouracil (5-FU), its analogues, as well as their combinations have been used with moderate efficacy in improving patients’ life [20,21]. Since 1997, gemcitabine has been accepted as a reference first-line therapy drug for patients with a good performance status [22]. Its advantage over 5-FU has been reported in different individual studies. In a comparative phase III study (*n* = 126) of single agent gemcitabine and 5-FU, a clinical benefit response was experienced by 23.8% of gemcitabine-treated patients compared to 4.8% of 5-FU-treated patients [22]. The median survival time was 5.6 and 4.4 months for gemcitabine and 5-FU-treated patients respectively, and the one-year survival rate was 18% for gemcitabine patients and 2% for 5-FU patients. All the results were statistically significant. Gemcitabine was also shown to substantially improve patients’ disease-related symptoms. Other phase II/III trials also reported a positive or partial positive response to gemcitabine, in the range of 5.4% to 12% [23,24] and median overall survival time ranging from 5 to 7.2 months [25]. One-year survival of 18% and median survival time of 6.2 months were reported in the successive study [26]. Importantly, besides grade 3 and 4 myelosuppression that was observed in around 30% of patients [26], lower systemic toxicity was attributed to gemcitabine treatment. Recently, CO-101, a lipid-drug conjugate of gemcitabine has been developed. The drug was designed to enter cancer cells independently of the human equilibrative nucleoside transporter 1 (hENT1) and therefore to overcome cancer resistance to gemcitabine; however, no significant difference in the efficiency of CO-101 and gemcitabine has been observed [27]. A modified version of gemcitabine (Acelarin) is currently under investigation in a phase III trial, with the aim to delay cancer cells’ resistance [28]. The addition of a phosphoramidate motif to gemcitabine was expected to diminish resistance acquired by PDAC cells after gemcitabine treatment. The data obtained so far showed that this modification increases the intracellular concentration of gemcitabine, mainly by ensuring its activity independently of nucleotide transporters.

### 3.2. Combination Therapies: Gemcitabine-Based Therapies

Following the positive results obtained with gemcitabine treatments, studies on more intensive and effective combination therapies composed of gemcitabine and different cytotoxic and biological agents have been developed. As previously mentioned, despite an acceptable toxicity profile and increased response rates, significant improvement in overall survival (OS) over single-agent gemcitabine was rarely observed [29,30,31,32,33]. However, when groups of patients were restricted to good performance status only, a survival benefit of combination treatment could be noticed [34,35,36]. In 2005, a combination of cisplatin, epirubicin, fluorouracil, and gemcitabine (PEFG) was tested for treatment of advanced PDAC patients [37]. A clear benefit in all efficacy parameters, together with moderately increased incidence of haematological adverse events, was observed. However, the small sample size diminished the value of these studies. In another study, 5-FU and fluoropyrimidine combination (S-1) showed a clinical benefit of the same efficiency as gemcitabine in metastatic patients [38]. Moreover, a combination of S-1 with gemcitabine showed improvement in most of the efficacy parameters and, despite the increased incidence of haematological toxicities such as neutropenia or thrombocytopenia, S-1/gemcitabine combination has become another viable option for a first line PDAC therapy, according to the results obtained from various randomised controlled trials in Asia [39]. It has been previously demonstrated that epidermal growth factor receptor (EGFR) is one of the molecules overexpressed in pancreatic cancer, playing an important role in carcinogenesis [40,41]. Moreover, its expression has been correlated with poor prognosis, metastasis, and sensitivity to chemo- and radiotherapy. Therefore, targeting this family of receptors presents a promising perspective for novel PDAC therapies and has been explored in a plethora of clinical trials. A Phase III trial examining the combination of gemcitabine and erlotinib (EGFR inhibitor) for the treatment of advanced and metastatic cancers showed moderate, but statistically significant improvement in both median survival rates (23% vs. 17%) and overall survival (6.2 vs. 5.9 months) [42]. Based on these results, gemcitabine/erlotinib combination received Food and Drug Administration (FDA) approval and became a preferred option for treatment of advanced, unresectable pancreatic tumours. Surprisingly, no correlation between EGFR expression and treatment efficiency has been noted (*p* = 0.4784) [43]. On the other hand, rash incidence, one of the adverse effects experienced by treated patients, seemed to correlate with patients’ positive response. Another gemcitabine-based combination, involving capecitabine, elicited significant prolongation of survival and became, together with erlotinib, one of the systemic treatment alternatives. Nevertheless, only patients with good PS responded positively to this treatment [34,44]. The effectiveness of capecitabine/gemcitabine combination applied as an adjuvant treatment was recently demonstrated in the European Study Group for Pancreatic Cancer (ESPAC)-4 trial. Combination of capecitabine with oxaliplatin (Cape-Ox) [45] as well as gemcitabine, docetaxel, and capecitabine (GTX) [46] are also used and restricted to good PS patients. Moreover, capecitabine’s superiority over gemcitabine as a radiosensitiser has been proposed in the selective chemoradiation in advanced localised pancreatic cancer (SCALOP) trial [47]. Other studies investigating combination therapy with gemcitabine showed very moderate or no significant improvement. Therapy using gemcitabine and platinum analogues (cisplatin or oxaliplatin) did not give clear results [30,32]. In some trials, the addition of cisplatin to gemcitabine had no effect on pancreatic cancer patients, whereas other studies showed an increase in median OS time (7.5 vs. 6 months) [32,33,48]. Table 1 lists former and current gemcitabine-based and combination therapies.

### 3.3. Abraxane and FOLFIRINOX: New Hope or Defeat?

Taxanes, such as docetaxel or paclitaxel, have been also considered for PDAC therapy. However, due to their poor solubility and consequently unsatisfactory delivery, their effectiveness was highly reduced. Nevertheless, a significant response to a combination of gemcitabine and albumin-bound paclitaxel (nab-paclitaxel, Abraxane) was observed in patients with advanced pancreatic cancer [49,50]. A synergistic effect of the drug combination was attributed to the improvement in the intratumoral delivery of both gemcitabine and paclitaxel, facilitated by fused albumin [51]. The effects of this combination treatment, in a phase III trial (*n* = 861), significantly surpassed the single-agent gemcitabine therapy in all tested parameters. The median OS time of 8.5 and 6.7 months was noted in Abraxane-gemcitabine and gemcitabine groups, respectively. A similar advantage was observed for progression-free survival (5.5 vs. 3.7 months) and one-year survival (35% vs. 22%). Unfortunately, the positive response to this therapy was accompanied by a considerable increase in occurrence of adverse events, including grade 3 or 4 neutropenia, leukopenia, neuropathy, febrile neutropenia, or fatigue [52]. Nevertheless, the increase in patients survival rates, at all time points, was a base for FDA approval and establishment of Abraxane-gemcitabine as the first-line therapy option for patients with advanced and metastatic pancreatic cancer. Its applicability for treatment of stage IV metastatic PDAC was also recently demonstrated in a case study, with increased quality of life and clinical response in a patient with a poor PS [53]. Interestingly, modification of the Abraxane administration regimen was proposed to improve its toxicity profile [54]. Recently, based on the proven advantageous and synergistic activity of its particular components [55,56,57,58], a multidrug combination (irinotecan, oxaliplatin, fluorouracil, and leucovorin) called FOLFIRINOX has been shown to be an effective first line therapy, especially for patients with metastatic pancreatic cancer. The anti-tumour effect in patients with advanced cancer was shown in a phase I trial [59] and confirmed in a phase II–III study, which explored patients’ response to FOLFIRINOX and single-agent gemcitabine [60]. The superiority of FOLFIRINOX over gemcitabine was recognised in all efficacy parameters, including OS (11.1 vs. 6.8 months), progression-free survival (PFS) (6.4 vs. 3.3 months), and one-year survival rate (48.4% vs. 20.6%), which presented statistically significant improvement. Unfortunately, the safety profile of FOLFIRINOX treatments was not favourable. The study showed increased incidence of grade 3 or 4 thrombocytopenia, neutropenia, febrile neutropenia, and diarrhoea, or grade 2 alopecia [60]. On the contrary, a significant reduction in the deterioration of quality of life was observed in patients treated with FOLFIRINOX compared to gemcitabine [61]. The positive response to FOLFIRINOX was also noted by a separate study conducted in India [62]. Despite its considerable toxicity, FOLFIRINOX is considered as a first-line option for patients with advanced and metastatic pancreatic cancer. However, its use is constrained to patients under the age of 75 and with good PS. To improve patients’ tolerance to the drug, modifications of FOLFIRINOX (e.g., mFOLFOX-folinic acid, fluorouracil, oxaliplatin- or FOLFIRI-folinic acid, fluorouracil, irinotecan) are currently being assessed [63,64].

Despite elevated adverse effects, the introduction of FOLFIRINOX and Abraxane to PDAC therapeutic repertoire brought new hope for patients and investigators. Considering that patients’ PS is one of the most important predictive factors, learning how to manage the toxicity of these multidrug regimens may further improve their feasibility. In addition, the failure of most of the gemcitabine-based combination treatments and the establishment of Abraxane as a new drug of reference in PDAC therapy makes it tempting to assume that the design of new clinical studies investigating Abraxane and FOLFIRINOX-based combination therapies might be a breakthrough in the improvement of the present grim perspective for PDAC patients.

## 4. Surgery—The Cornerstone of PDAC Therapy

**Figure 1:**
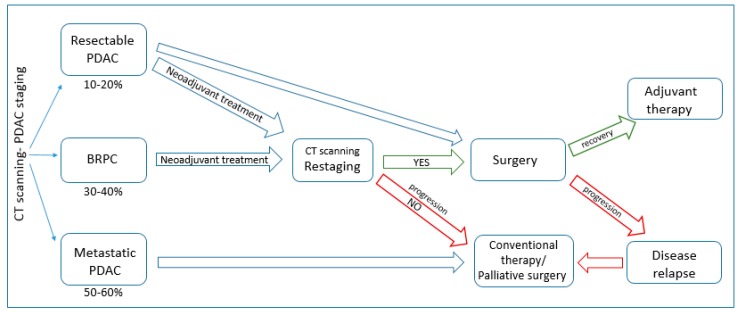
Schematic model of therapeutic strategies for diagnosed pancreatic ductal adenocarcinoma green—successful procedure; red—failed procedure.

Considering the lack of definite survival benefit presented by conventional chemotherapy, complete resection followed by adjuvant treatment remains the only realistic curative option for PDAC patients. In general, the operability status is dictated mainly by the extent of venous involvement. However, the choice of surgery and its extent is imposed not only by the tumour localization and extension, but also by the surgeon’s expertise and by the patient’s performance status (PS), which is one of the major prognostic factors. For patients that are eligible for resection (resectable, borderline resectable), available surgical options are: pancreaticoduodenectomy (head/body of the pancreas and nearby organs are removed), distal pancreatectomy (tail, body and spleen), total pancreatectomy (whole pancreas and nearby organs) or palliative surgery (stent or bypass), which may alleviate symptoms of biliary and gastric outlet obstruction [66]. Pancreaticoduodenectomy, introduced by Whipple and Kausch at the beginning of 20th century, is a three-step procedure of exploration, resection and reconstruction. It is currently a safe procedure and results in low mortality and morbidity [67]. Significantly worse postoperative recovery and outcome has been demonstrated after total pancreatectomy, which is reserved for few indications, mainly because of metabolic imbalance [67]. The extent of the resection has been widely discussed over the last years; however, none of the procedures showed significant superiority over the standard pancreaticoduodenectomy. One of the most important factors for prognosis of postoperative survival and surgery success is R0 resection, in which histologically free margins are detected [68]. In R1 and R2 resections, microscopic and macroscopic tumours are still visible at the margins and correlate with reduced survival [16]. In borderline resectable and locally advanced tumours, vascular resection and reconstruction of superior mesenteric vein/portal-vein (SMV/PV) should be considered. It has been confirmed in a series of studies that SMV/PV resection and, in some cases, arterial resection should be performed in order to achieve R0 resection without reducing patient’s survival compared to standard PD and so achieving similar outcomes for all resectable patients [69,70]. For patients with tail and body cancers with venous encasement, extended distal pancreatectomy with resection of celiac artery has been proposed, however the small number of studies conducted on this procedure limits the determination of its survival benefits [71,72,73]. In some cases, splenectomy must be performed as well; however, there is still controversy over splenic preservation and its impact on patient’s overall outcome [67]. Tumour size is one of the most important independent prognostic factors [74]. It has been demonstrated that larger tumours can be associated with higher venous involvement and thus with high probability of microscopically positive resections (R1) [70]. Higher blood loss during surgery, which is another prognostic factor, has been also reported during resection of larger tumours [75]. Unfortunately, only in 2% of diagnosed patients, tumours smaller than 2 cm in diameter, which is the statistical cut-off, are detected. Another survival factor is the ratio between examined and negative lymph nodes, described as lymph node ratio (LNR), which may give more insight into the extent of the metastatic disease [15]. There are some discrepancies on whether extended lymphadenectomy has any benefit in terms of survival over standard lymphadenectomy [76]. Nevertheless, it has been shown that it considerably increases both R0 resection rate and survival, which highly depends on the number of resected and negative lymph nodes [77,78]. However, the jury is still out on the minimal number of lymph nodes that should be resected and examined to properly assess the prognosis. Despite the low percentage of patients undergoing surgery, the chance of survival for surgical patients has significantly increased in the last few decades. Regardless of considerably high postoperative complications, the mortality rates do not exceed 5% [79]. The effectiveness of surgery and patients’ long-term survival depends partially on lymph-node infiltration but also on the surgeon’s expertise and the number of operations performed by the hospital. Unfortunately however, even after successful resection the median survival time is 20 months, with 25% five-year survival rate [74]. The majority of resected patients suffers from tumour recurrence (~40%) within 6–24 months post-surgery [80], highlighting the necessity for preoperative/postoperative therapies in order to achieve more effective treatments. Therapeutic regimen options for PDAC patients are presented in Figure 1.

## 5. Neoadjuvant and Adjuvant Therapies

Surgery followed by adjuvant therapy has been shown to provide slight, but significant survival benefit for non-metastatic patients in several phase III studies. Thus far, gemcitabine and 5-FU-based postoperative chemoradiation has been considered as standard of care, improving the median OS time for 2–5 months [81,82]. However, adjuvant therapy remains a controversial field, with results obtained in clinical trials ranging from definite survival benefit [83] to negative impact on patients’ OS [82]. In addition, almost 60% of resected patients present early tumour progression or prolonged recovery, disabling planned postoperative treatment. Therefore, if no distant metastasis has been detected during cancer diagnosis and staging, the recommended first line treatment is neoadjuvant chemotherapy. This therapy aims to enhance drug delivery and tumour oxygenation and minimise tumour burden, which may result in downstaging and more definite surgical resection [84] and reduce the risk of tumour implantation during pancreatectomy [85]. Preoperative treatment might also avoid the delay between the diagnosis and the start of postoperative treatment, usually caused by patient’s prolonged recovery, and enable treatment of early micrometastases [86]. In addition, higher number of patients may benefit from this treatment compared to patients profiting from adjuvant therapies, which sometimes need to be postponed or cancelled due to postoperative complications, prolonged recovery or early recurrence [87,88]. Cancer chemo-sensitivity might be also determined. However, neoadjuvant treatment also raises several concerns, such as disease progression during preoperative treatment or possible increase in surgical complications. Nevertheless, no difference in morbidity or mortality has been observed so far between patients subjected or not to preoperative treatment [89]. On the other hand, neoadjuvant therapy may avoid unnecessary surgery in patients, whose disease progressed during the treatment, therefore selecting a subpopulation of patients that might benefit from further therapy [90,91]. However, this aspect remains debatable. Different approaches for preoperative therapy, such as chemoradiation, chemotherapy followed by radiotherapy or chemotherapy alone have been considered, even though none of the strategies showed considerable superiority. The optimal therapy, its duration and the most adequate time for its initiation still need to be determined. Thus far, there has also been no consensus on the advantage of preoperative over postoperative treatment, making the use of this therapy controversial.

### 5.1. Neoadjuvant Therapy in Resectable Patients

Application of neoadjuvant therapy for localized resectable tumours remains controversial since it delays the surgery and may result in disease progression. On the contrary, preoperative therapy of resectable patients reduces the risk of intraoperative tumour spillage, sterilizes lymph nodes and may improve patients’ response to postoperative treatment. It has been shown in different independent studies that, in patients who showed no progression after applied therapy, higher rate of R0 resections, lower recurrence and better survival are observed [92,93,94,95]. As for the choice of therapeutic regimen, different strategies have been studied, most of them being gemcitabine-based. Twenty-three-month survival and 66% R0 resection rate have been shown after gemcitabine-based radiotherapy, demonstrating feasibility of neoadjuvant therapy in resectable tumours [96]; however, the single arm design of this study impairs its proper evaluation. Gemcitabine combined with cisplatin presented significant increase in the resection rate (70%) compared to gemcitabine alone (38%) when applied in preoperative settings [97]. Phase II trial of gemcitabine and cisplatin is currently ongoing. Another study on the same combination proved 71% R0 resection rates and 26.5 months survival for resected patients [98]. Gemcitabine applied with oxaliplatin and radiation therapy resulted in overall survival (OS) of 18 months after successful surgery [99]. Considering modest improvement with gemcitabine/cisplatin combination in resectable tumours, the same modality followed by gemcitabine-based chemoradiation has been tasted in phase II trial. However, 66% resectability and OS of 17.4 months did not give ground to continue the study [100]. Neoadjuvant vs. post-operative radiation for resectable patients has been evaluated in a large retrospective study, showing slight benefit for the preoperative group (23 vs. 17 months) and definite advantage over untreated patients (12 months) [101]. However, the results of meta-analysis in several studies contradicted these results, showing no benefit in any parameter [102]. Interestingly, radiochemotherapy was claimed to be superior to monotherapy treatment by Gillen and colleagues [103]; however, only marginal benefit of preoperative gemcitabine with or without radiation has been reported in another study. 5-FU based chemoradiation has been widely applied as neoadjuvant treatment for resectable tumours [104]; anyhow, its efficacy is far from being optimal. It also showed considerable toxicity, with 32% of patients requiring hospitalization in one of the conducted studies [92]. Modification of applied treatment schedule (30 Gy instead of 50.4) helped to reduce the toxicity, however obtained results were not promising [105]. Similarly, detrimental effect (61% resection rate) has been reported in retrospective meta-analysis of 5-FU and cisplatin combination. Paclitaxel and radiotherapy have been also evaluated; however, the results (34% R0 resection) did not show any advantage over gemcitabine or 5-FU-based treatments [106]. Taken together the results show that no consensus can be reached on the applicability of neoadjuvant treatment for resectable tumours and no difference between preoperative and postoperative therapy has been reported in terms of survival benefit. In addition, limited number of cases and mostly retrospective studies restrain proper analysis of conducted studies. However, available data and theoretical reasoning justifies its use.

### 5.2. Neoadjuvant Therapy in Borderline Resectable and Locally Advanced PDAC

There is considerable inconsistence in the definition of borderline resectable pancreatic cancer (BRPC), hampering proper design of studies. BRPC is a “marginally” resectable lesion with no distant metastasis, however showing perivascular fat plane absent over 180 degrees of SMN and PV [107,108]. Callary and colleagues summarized this criteria and additionally stated that no CT evidence of vascular encasement should be seen in resectable tumours [109]. Recently, this concept was extended by the MD Anderson Cancer Center (MDACC), including tumour biology, anatomic relationships and patient’s condition to specifically classify diagnosed disease [8]. Therefore, borderline resectable tumours have been divided into three categories: (A) anatomically defined BRPC; (B) possibility, but not diagnostic staging for micrometastatic disease; and (C) marginal PS, but susceptible for surgery. Definition of BRPC has developed over the years; however, its determination in clinical practice is still challenging. There are some difficulties to demarcate BRPC patients and those with locally advanced tumours, which highly influence the response and therefore, provide inconsistent data. Nevertheless, posing the risk of incomplete, margin-positive resection, BRPC patients seem ideal candidates for neoadjuvant therapy in order to complete successful margin-negative resection [8,89]. However, inconsistencies in proper tumour classification, as well as difficulties in determining downstaging effects before resection, caused by dense stroma and the presence of pancreatitis, influence the clinical interpretation of results and therefore have an impact on the proper assessment of its efficacy. As an example, it has been reported by Hoffman and colleagues that a response rate of less than 10% has been detected by CT scan after preoperative treatment, whereas tumour response of 71% has been stated after surgery [110]. Similarly, White and colleagues suggested that dependence on CT imaging for the determination of tumour resectability may deprive around 12% of patients of the chance for R0 resection [111]. Moreover, in another study, despite only 0.8% of patients presenting a radiologically restaged disease after the preoperative treatment, 80% of the studied population underwent surgery with 95% of R0 resections, proving the inapplicability of radiographic imaging for the evaluation of post-treatment outcomes [112]. The inaccurate assessment of treatment efficacy may result from fibrotic scar changes caused by neoadjuvant chemoradiotherapy (neoCRT), which may cause false negative results. Aforementioned results demonstrated that CT scanning is of little clinical value in assessment of tumour response to therapy and proposed that, provided good PS and lack of metastasis, tumour resection should be performed regardless of radiographic evaluation of preoperative treatment efficacy. Margin status should be considered as a more appropriate indicator of the response to neoadjuvant treatment.

Thus far, there have been very few prospective studies assessing the advantage of neoadjuvant therapy in borderline resectable PDAC. In the first one, carried out in 2001 on 15 patients, 5-FU and radiation were applied in order to downstage the tumour and allow surgery. Sixty per cent of patients were able to have a complete resection with negative margins, leading to the conclusion that chemoradiation may successfully improve resectability in “marginally resectable” patients [113]. Another, multi-centre prospective study compared different neoadjuvant modalities in 21 borderline patients. Ten of them received gemcitabine-based chemoradiation and the remaining 11 were subjected to induction chemotherapy (gemcitabine/cisplatin/5-FU) followed by 5-FU based radiation. Regrettably, the study had to be terminated due to lack of significant improvement; however, it showed that both regimens are well tolerated and have similar effectiveness [114]. Unfortunately, the majority of studies are of retrospective nature, most of them showing good tolerability and modest efficacy in resectability improvement. Different strategies for neoadjuvant therapies of BRPC have been applied. Gemcitabine/capecitabine, gemcitabine/oxaliplatin [115] and 5-FU based treatments have been tested and showed modest improvement in resectability; e.g., 40% of gemcitabine/capecitabine-treated patients was able to receive surgery, with 82.3% R0 resections [116]. Gemcitabine and S-1 combination has been also evaluated for both: resectable and BRPC tumours, achieving 74% R0 resection rate. A two-year survival for almost 46% of patients and median OS time of 35 months after completion of surgery appear to be encouraging [117]. Gemcitabine and docetaxel combination (NeoGemTax) applied in neoadjuvant settings allowed for R0 resection in 87% of patients, initially stated as borderline resectable or unresectable [115]. Radiotherapy (RT) has provided slightly better results in terms of surgery rates (74% vs. 61%) and decreased recurrence [87], however no superiority over chemotherapy has been observed in terms of survival. Concurrent chemoradiation has brought considerably encouraging results. Several studies have tested full dose chemotherapy (e.g., gemcitabine, S-1 or capecitabine) combined with full dose radiotherapy [118,119]. A Japanese retrospective study on gemcitabine-based radiation therapy demonstrated 92% of R0 resections that could be completed for patients receiving treatment, compared to 52% of untreated patients. Consequently, higher long term survival has been reported (59.4% two-year survival) [120]. Evans and colleagues proved the superiority of gemcitabine over 5-FU-based RT and a median OS time of 34 months for patients subjected to successful surgery [96]. Gemcitabine-based therapies, combined with radiation, were explored in multiple studies showing promising results. Taken together, data showed that the resectability rate ranged between 24–100%; however, the vast majority of those patients (67–100%) were able to receive the R0 resections, demonstrating the efficacy of neoadjuvant therapy [121]. Chemotherapy (e.g., gemcitabine, 5-FU or gemcitabine/cisplatin) followed by radiotherapy has been also considered, with increased possibility to perform pancreatoduodenectomy in patients subjected to preoperative treatments [121]. In a more recent study review, 57 borderline resectable patents were treated with Gemzar, Taxotere and Xeloda, followed by radiation. The results of this study showed that 56% of patients were able to receive surgery, with almost 97% of them having R0 resection [122]. In general, after the analysis of available data, it can be observed that around 30% of borderline resectable patients subjected to neoadjuvant treatment may undergo surgery and, in these cases, mostly complete R0 resections are performed. This shows promising perspectives; however, the retrospective character of most of the studies and the low number of patients highly hinder drawing proper conclusions.

Although less promising and challenging, preoperative treatment followed by surgery has also been considered for locally advanced pancreatic cancer (LAPC). It has been demonstrated in several studies that preoperative treatment applied to LAPC patients decreased lymph node involvement, which may be considered as a positive predictor of survival benefits [118]. Improvement in OS for LAPC patients has been demonstrated in few studies [123], however most of the reports showed contradictory results. One of the meta-analysis of patients with advanced PDAC tumours demonstrated that an average 33.2% of analysed patients underwent surgery, with 79.2% of R0 resections after completion of preoperative treatment. Results were comparable with these of initially resectable patients, whose R0 resection rates were 82.1% [103], demonstrating the feasibility of this approach and underlining the potential of neoadjuvant treatment to increase resectability, even for locally advanced tumours. Gemcitabine-based combinations, such as gemcitabine and oxaliplatin (NeoGemOx) [115], gemcitabine and capecitabine [47,116], PEFG/PEXG (cisplatin, epirubicin, 5-FU/capecitabine and gemcitabine) or PDXG (docetaxel replacing epirubicin) [124] showed promising results in converting tumours to resectability and increasing the number of patients subjected to surgery, demonstrating the feasibility of subjection of LAPC to neoadjuvant therapy. FOLFIRINOX-based regimens recently emerged as new restaging strategy, significantly increasing resection rates [123,125,126]; however, more prospective studies are necessary to fully evaluate their efficacy. Additionally, a case study of a patient with locally advanced PDAC demonstrated that the nab-paclitaxel/gemcitabine combination followed by FOLFIRINOX resulted in tumour remission and completion of R0 resection [127]. No disease progression was observed 18 months following the completion of the therapy. This case demonstrates the potential benefit of neoadjuvant therapies for locally advanced tumours and makes it worth considering further exploration.

Overall, it has been shown in a recent meta-analysis that the median survival time of 18–20.5 months could be achieved in initially unresectable patients [8]. Importantly, it has also been demonstrated that neoCRT significantly reduced local recurrence compared to adjuvant treatments (34% vs. 5%) [93]. Unfortunately, another large retrospective analysis demonstrated that around 65% of preoperatively treated and resected patients had tumour recurrence, with 40% of them developing distant metastasis [81]. Therefore, down-staging neoadjuvant therapy combined with treatment targeting micrometastasis, undetectable at the time of diagnosis, could improve this grim prognosis. Following the success of nab-paclitaxel and FOLFIRINOX in metastatic disease, new strategies involving combination treatments based on these drugs have also been considered in neoadjuvant/adjuvant settings [128,129]. Currently, FOLFIRINOX-based preoperative therapy is being tested for improved efficacy, mainly in down-staging tumour burden, as well as targeting micrometastasis.

### 5.3. Adjuvant Therapy in PDAC

Once completing successful surgery, post-resection adjuvant therapy is subsequently applied to reduce the risk of relapse. 5-FU-based chemoradiation has been claimed to improve survival up to 10 months in the Gastrointestinal Tumour Study Group (GITSG) trial [130]. However, the same combination failed to demonstrate any benefit in the subsequent European Organisation for Research and Treatment of Cancer (EORTC) trial [131], which was attributed to suboptimal RT dose (40 Gy instead of 50) and 20% of patients failing to receive full chemotherapy treatment. Gemcitabine-based adjuvant therapy has been explored in the Charité Onkologie CONKO-001 trial [81]. A significant improvement in all evaluated parameters (PFS, OS) could be observed, providing evidence of the benefit of gemcitabine-based post-operative treatments. In addition, the durable effect of investigated regimen has been demonstrated in a follow-up study (10-year survival of 14.3% compared to 5.8% for non-treated group) [132]. Chemoradiation with the use of gemcitabine/docetaxel/capecitabine (GTX) followed by 5-FU/RT is also considered [133]. Disappointing results were delivered by the ESPAC-3 trial, comparing 5-FU and gemcitabine-based treatments, which did not prove any benefit for the gemcitabine arm [133]. However, median survival times in both arms surpassing 20 months confirmed efficacy of both regimens. Similar results were provided in the radiation therapy oncology group RTOG 9704 trial, in which the improvement in the gemcitabine arm did not show statistical significance [134]. S-1 has been also compared with gemcitabine for adjuvant treatment in the Japan Adjuvant Study Group of Pancreatic Cancer JASPAC-01, showing an encouraging five-year survival rate of 44.1% in the S-1 group compared to 24.4% for the gemcitabine treated patients [135]. Based on this data, S-1 should be considered as another standard of care; however, the analysis was restricted to Asian population. No survival benefit has been demonstrated with combination of erlotinib and gemcitabine in adjuvat setting (CONKO-005). No difference in PFS (11.6 months for both groups) or OS (24.6 months for erlotinib/gemcitabine and 26.5 months in gemcitabine arm) was observed. However, an estimated long-term effect in favour of the erlotinib group (five-year survival of 28% vs. 19% for gemcitabine) gives ground for further exploration of this approach [136]. Phase II and phase III trial assessing the role of erlotinib in addition to gemcitabine in adjuvant setting and determining the efficacy and safety of concurrent fluoropyrimidine after gemcitabine-based or non-gemcitabine based adjuvant therapy is currently conducted by the Radiation Therapy Oncology Group (RTOG) foundation [137]. Several trials are also exploring the use of FOLFIRINOX and Abraxane following surgery. Studies comparing adjuvant gemcitabine and mFOLFIRINOX (NCT01526135), adjuvant gemcitabine and neoadjuvant and adjuvant FOLFIRINOX (NCT02172976), as well as pre-operative and adjuvant FOLFIRINOX (NCT01660711) are currently ongoing. Similarly, phase II study of the nab-paclitaxel and FOLFIRINOX is currently ongoing (NEOLAP; NCT02125136). The Adjuvant Pancreatic Adenocarcinoma Clinical Trial (APACT) trial (NCT01964430) is also evaluating nab-paclitaxel and gemcitabine vs. gemcitabine alone to treat resected patients.

Taken together, discrepancies exist in the evaluation of the efficacy of neoadjuvant and adjuvant therapies. Their benefit has been claimed in a retrospective study (1999–2006), in which preoperative chemotherapy followed by radiation resulted in 78% of patients completing restaging, 53% resection rate and overall better clinical outcome [103]. However, another retrospective analysis of PDAC resections (1992–2011) showed no difference in resection margins between untreated patients and those subjected to neoadjuvant treatment [138], undermining the concept of neoadjuvant therapies. This lack of consensus is mostly due to no unequivocal definition of borderline resectable cancers, small collection of cases and limited number of prospective studies, impeding proper evaluation and interpretation of the results.

Nevertheless, many clinical trials are still ongoing in order to combine the best neoadjuvant agents with postoperative adjuvant therapies, hoping to obtain more prominent improvements in the survival of patients with resectable or borderline resectable tumours. Neoadjuvant FOLFIRINOX and postoperative gemcitabine [139] are presently under investigation in a multi-institutional Alliance trial (NCT01821612). Thus far, no severe adverse events, precluding from completion of surgery, have been reported. Sixty-eight per cent of patients underwent surgery, with 93% R0 resection rate. At the time of the initial evaluation, 82% of patients were still alive, with median post-treatment survival time of 10 months. Nab-Paclitaxel/gemcitabine combination has been recently explored in the context of preoperative therapy for both borderline resectable and locally advanced tumours. The NEONAX (NCT02047513) and the GAIN-1 (NCT02210559) studies are currently under investigation. Immunotherapy (GVAX vaccine, CD40 antagonists), neoadjuvant capecitabine (CAPOXIRI; NCT01760252) or studies of different FOLFIRINOX regimens are currently ongoing [140].

## 6. Targeted Therapies—A New Prospect for PDAC Treatment?

**Figure 2:**
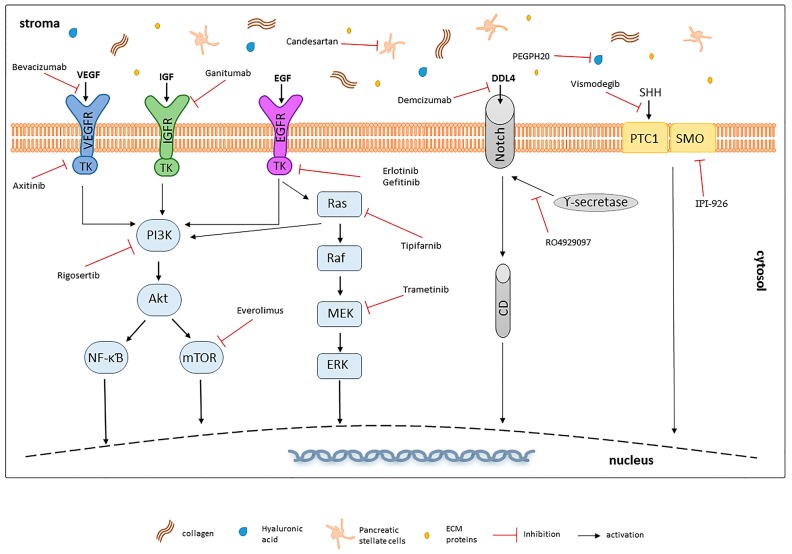
Comparison of selected targeted therapies in as anti-pancreatic ducal adenocarcinoma (PDAC) approach.

As aforementioned, pancreatic cancer presents high heterogeneity in terms of mutational landscape of crucial signalling pathways. Most of pancreatic tumours (around 95%) carry *RAS* mutations. The most frequent among them are *KRAS* alterations (85%), which mainly consist of substitution of G12, resulting in a constitutively active protein [141]. *KRAS* mutations have been recognized as the earliest event in PDAC initiation (PanIN1); however, this is not a sufficient requisite for cancer onset and its progression [142]. During tumour development, *KRAS* alterations accumulate, together with other mutations that pile up progressively. Other common mutations include inactivation of cyclin-dependent kinase inhibitor 2 (*CDKN2*) (in around 90% of PDAC cases) and mothers against decapentaplegic homolog 4 (*SMAD4/DPC4*) (~55%), *BRCA2*, MutL homolog 1 (*MLH1*) or protease, serine 1 (*PRSS1*) alteration. Furthermore, 50–70% of PDAC cases carry mutation in the tumour protein 53 (*TP53*) gene, which occur at later stages of PanIN, contributing to the malignant progression of PDAC rather than its initiation [143]. Such variety of accumulating mutations results in the dysregulation of a plethora of signalling pathways playing a vital role in many crucial processes including apoptosis, cell proliferation and differentiation. Overall, around 60 mutations in 12 different signalling pathways accompany the development of aberrant ducts in PDAC [144]. Among many, changes in Hedgehog, Notch, Wnt, transforming growth factor beta (TGF-β) and RAS/MAPK/PI3K, JAK-STAT pathways, which are normally responsible for the correct development of the pancreas, are recognized as main contributors in PDAC progression [145,146,147]. In addition, crucial molecules and pathways from both the tumour itself and the surrounding stroma, such as EGFR-mediated pathways, proangiogenic or embryonic pathways influence PDAC resistance to therapy and correlate with poor prognosis. Considering the wide variety of signalling pathways dysregulated in pancreatic cancer and triggering its progression, targeted therapies have emerged as a possibility to augment available therapeutic strategies (Figure 2). This approach has been successfully implemented in the treatment of different solid tumours, with imatinib mesylate (Gleevec) being the first FDA approved targeted treatment of metastatic gastrointestinal tumours in 2002 [148]. Since then this therapeutic approach has been widely used and many targeted drugs for e.g., colorectal, melanoma or non-small lung cancer have been approved [149,150]. However, due to the heterogeneous nature of pancreatic cancer and complex stromal interactions, most of the targeted therapies failed to exhibit any clinical benefit compared to standard treatment. The only exception was erlotinib, an epidermal growth factor receptor (EFGR) inhibitor that, in combination with gemcitabine, showed a moderate but statistically significant (two weeks) improvement in patients’ survival [42]. Although many of the studies on targeted PDAC therapies showed promising results in preclinical or clinical settings, most of them failed during phase II/III trials (Table 2). Nevertheless, numerous phase I/Ib studies are still ongoing with many of them showing encouraging results, enabling to move on to phase II/III trials.

### 6.1. Targeting Growth Factor Receptors

EGFR belongs to ErbB family of receptors, containing a tyrosine kinase domain, which activation is involved in regulation of key processes such as cell cycle, cell survival and differentiation through activation of multiple downstream signalling pathways, including RAS/PI3K/Akt or MAPK/ERK. EGFR pathways are over-activated in PDAC as a consequence of high receptor density, overexpression of ligands or EGFR activating mutations [151]. Considering the high prevalence of EFGR mutations in pancreatic cancer patients and the success of erlotinib, an adenosine triphosphate (ATP) competitor for binding to tyrosine kinase (TK) domain, as a PDAC therapeutic, other molecules targeting this pathway have been intensely tested. Both antibodies blocking EGFR activation and inhibitors of tyrosine kinase domain of the receptor have been evaluated. However, most of them failed to show any improvement over the standard treatment. As an example, cetuximab, an EGFR-binding monoclonal antibody, showed promising phase I results in combination with capecitabine but revealed no statistical significance in survival benefit in further studies [152]. Gefitinib treatment combined with gemcitabine was also evaluated and resulted in 1 year survival rate of 27% and median survival time of 7.3 months [153]. Although encouraging, gefitinib has been considered not as promising as erlotinib. Therapies designed for patients harbouring human epidermal growth factor receptor 2 (*HER-2*) mutations emerged as another possibility. It has been demonstrated that more than 10% of PDAC patients overexpress HER-2 protein and its expression has been correlated with patients’ poor survival [154]. Therapy of HER-2 positive patients with capecitabine and trastuzumab, though promising, was unsuccessful in phase II clinical trials [155]. The main drawback of this study was the small number of patients harbouring *HER-2* alterations. After getting FDA approval for chemotherapy, lapatitib has been also tested in combination with gemcitabine for pancreatic cancer patients; however, the results showed only moderate improvement, with a median survival time of four months [156]. Lapatinib/capecitabine combination has been also tested as second-line therapy for pancreatic cancer. Although the treatment was well tolerated and provided improvement for a subset of patients, the limited number of participants impairs evaluation of its clinical benefit [157]. Nimotuzumab (anti-EGFR monoclonal antibody) [158] and afatinib (TK inhibitor) [159] also showed encouraging results in preclinical or clinical studies and their therapeutic application is currently under evaluation. Insulin-like growth factor 1 receptor (IGF1R) is also highly overexpressed in pancreatic cancer and its excessive activation leads to boosted stimulation of downstream pathways, increasing cell proliferation and survival [160]. Several drugs targeting these molecules, especially monoclonal antibodies ganitumab and cixutumumab have been evaluated; however, no statistically significant improvement of survival was observed [161]. Similarly, a study on the combination of ganitumab and gemcitabine failed to show significant benefit over the single agent gemcitabine in phase III clinical trial causing the closure of the study [162].

### 6.2. KRAS Pathways Inhibition

*KRAS* mutations are widespread in pancreatic cancer, with more than 90% of diagnosed patients having mutated *KRAS* gene. Membrane-bound guanosine triphosphate hydrolase (GTP-ase) protein encoded by this gene is activated by the family of EGFRs and induces signalling involved in a plethora of cellular functions. When mutated, KRAS gains oncogenic activity and is maintained in a constitutively active state, continuously inducing downstream signalling pathways (MAPK/ERK, PI3K/Akt) contributing to increased proliferative signals, invasiveness and inhibition of cell apoptosis. Although the idea of a KRAS inhibition raised a lot of hope, its direct targeting did not bring the expected results. Therefore, strategies targeting proteins along the RAS signal transduction pathway have been widely explored. For example, tipifarnib, an inhibitor of farnesyl-transferase (an upstream effector of RAS, essential for its activation) was studied in combination with gemcitabine but unfortunately, showed no superiority over standard therapy in phase III trial [163]. Another strategy is blocking KRAS downstream signalling molecules, such as MAPK pathway, which activation is observed in later stages of pancreatic cancer and favours cancer development. However, MEK targeting, with selumetinib combined with capecitabine [164] or trametinib/gemcitabine combination [165], was not able to increase OS or provide statistically significant results. Nevertheless, taking account of the promising results obtained in preclinical studies, ERK inhibition is still explored as a potential pancreatic cancer treatment. A combination of trametinib and GSK2256098 (focal adhesion kinase, FAK inhibitor) is planned to be tested and a proposed study is currently recruiting participants (NCT02428270). Ulixertinib BVD-523, an ERK inhibitor, is also currently tested in combination with gemcitabine/nab-paclitaxel in phase Ib trial [166]. Another crucial pathway in pancreatic cancer is PI3K signalling, that is activated in response to EGFR induction, and in turn, triggers activation of several downstream targets such as Akt, pS6 or mTOR, influencing cell survival, metabolism and proliferation [167]. Therefore, PI3K signalling inhibition represents another possibility for PDAC therapy. A combination of gemcitabine and rigosertib, a Ras mimetic and small molecule inhibitor of PI3K, has been evaluated; however, it failed to enhance patients’ response when combined with gemcitabine [168]. Data from everolimus and sunitinib (mTOR inhibitors) studies suggested promising results, incrementing the progression-free survival time (from ~5 to 11 months) [169,170], potentially improving prognosis for a selected groups of patients. A combination of everolimus and capecitabine has also been tested resulting in 8.9 months OS [171]. Being a single arm study, the impact of everolimus on patients’ response is hard to determine. Nevertheless, considering previous results of capecitabine monotherapy showing 5.9 months survival, the achievement of 8.9 months seems encouraging. However, the differences in the study’s design and patients’ population make this assumption arguable. Likewise, disappointing results were obtained in other phase II studies, in which everolimus or temsirolimus were used to inhibit PI3K/Akt/mTOR pathways [172,173]. Another mTOR inhibitor, PBI-05204 (NCT02329717), is currently tested for patients with stage IV pancreatic cancer. Moreover, it is considered that combining PI3K and MEK inhibitors may have a potential synergic activity [174].

### 6.3. Targeting Angiogenesis

Angiogenesis is a pivotal process required for tumour growth and metastasis. Therefore targeting the mechanisms regulating this process seems to be a tempting strategy to reduce cancer progression. Among many factors, vascular endothelial growth factor (VEGF) and its receptor have been mostly studied in the context of the abovementioned process [151]. It is claimed that therapy against those molecules, although not effective in terms of modulation of cancer cell proliferation in vitro, may reduce proliferation of endothelial cells, decrease infiltration and metastasis in vivo. However, studies investigating the anti-angiogenic agents axitinib (inhibitor of VEGFR, mast/stem cell growth factor receptor SCF) and platelet-derived growth factor receptor PDGFR tyrosine kinases) [175,176] or Avastin (bevacizumab, a VEGF-A inhibitor) [177] did not exhibit positive and statistically significant results. Due to unmet primary endpoint of OS, Pfizer had to discontinue its study on axitinib combined with gemcitabine [178]. Likewise, phase II study or sorafenib (Raf kinase, VEGF-R2/R3 and PDGFR-β oral inhibitor) alone or in combination with gemcitabine did not exhibit promising activity in metastatic patients [179]. Similarly, addition of aflibercept (a recombinant protein targeting VEGF signalling) to gemcitabine, although promising in pre-clinical studies, did not improve patients’ OS and resulted in an increase of the incidence of adverse effects [180]. Likewise, a study on necuparanib and nab-paclitaxel/gemcitabine, although initially promising, had to be terminated due to lack of expected efficacy [181]. At present, phase II trial of the novel anti-angiogenic agent TL-118 (NCT01509911) is being assessed.

### 6.4. Other Targets

One of the most encouraging results so far has been obtained from JAK-STAT pathways inhibition studies, especially in tumours with an inflammatory microenvironment. The role of JAK-STAT pathway in cell proliferation migration and apoptosis has been widely elucidated. Increased expression of the members of these two pathways in PDAC has been shown by gene-expression analysis [182] and they have been shown to directly contribute to the initiation and progression of pancreatic cancer. JAK1 and JAK2 inhibition with a capecitabine and ruxolitinib combination did not show significant benefits in the survival of untreated patients. However, in patients resistant to gemcitabine, the combination showed improvements in performance status and pain management [183] and phase III studies of this combination are currently ongoing [184]. A phase III study evaluating the Janus kinase inhibitor momelotinib in combination with nab-paclitaxel/gemcitabine has just terminated (NCT02101021) and the results are expected to be published. The importance of Notch pathway in PDAC is also well known, and its role in chemoresistance was highlighted in various reports [185,186]. It has been shown that its inhibition, i.e., through anti-DDL4 antibodies (tarextumab or demcizumab) combined with gemcitabine, exhibited anti-tumour activity and indicated a possible reversal of chemoresistance, mainly by targeting pancreatic cancer stem cells [187] and therefore showing a therapeutic potential. However, although after a promising phase I outcomes, the Yosemite trial, evaluating the combination of demcizumab and gemcitabine/Abraxane had to be discontinued due to unmet primary endpoint of PFS [188]. Moreover, an interim OS analysis failed to show any benefit over the Abraxane arm. Recent exciting results have been obtained with gemcitabine and MK-0752 (an inhibitor of γ-secretase, the cleaving enzyme in Notch-mediated cascade), although further studies are needed [189]. Another γ-secretase inhibitor, RO4929097, has been tested in phase II studies, in which good tolerance and moderate OS response was reported; however, the limited cohort of 18 patients limits proper assessment of this study [190]. Interestingly, it has been suggested that combined targeting of both JAK and Notch pathways surpasses their individual inhibition, however the effect of that approach on patients’ outcome is still to be determined.

Poly ADP-Ribose pathway (PARP) presents another possibility for targeting PDAC. These enzymes are activated in response to DNA damage and it has been shown that patients with a defective DNA recombination pathway may positively respond to PARP inhibitors [191]. Moreover, BRCA mutations, impairing DNA repair, might be also targeted by those compounds. Therefore, many clinical trials targeting this pathway are currently ongoing. Olaparib is an oral poly (ADP-ribose) polymerase inhibitor, which has shown promising activity in different cancers bearing BRCA mutations [192]. Olaparib is currently being tested in a phase III trial for patients with BRCA mutated pancreatic cancer (NCT02184195) and combination of gemcitabine/cisplatin with another PARP inhibitor, veliparib, is also being evaluated [193,194]. Tumour suppressor *TP53* is another gene highly mutated in PDAC progression. Its normal activity is essential for cell apoptosis, cell metabolism and DNA damage repair, therefore its deactivation highly contributes to the development of a plethora of malignancies [143]. Study of p53 targeting molecule, SynerGene Therapeutics 53 (SGT-53), is being currently tested in combination with nab-paclitaxel/gemcitabine (NCT02340117).

### 6.5. Targeting Tumour–Stroma Interactions

One of the reasons for the dismal prognosis of PDAC is a high chemoresistance caused by the huge genetic heterogeneity and plasticity of PDAC tissues. An additional factor contributing to cancer resistance is the formation of a dense, diffuse stroma called desmoplasia [5]. Pancreatic stellate cells (PSCs), fibroblasts, blood vessels and proteins form a dense environment through the expression of multiple molecules (e.g., chemokines, EGFs, Cox-2) and interact with cancer cells, influencing tumour progression and invasion [7]. Other than forming a dense barrier around the tumour, the desmoplasia is also responsible for poor vascularisation of tumours and consequently, causes nutrient depletion as well as impairs drug delivery to cancer cells [6]. Therefore, it has been shown that, by formation of a cancer promoting environment, cancer stromal cells influence PDAC development. The cross-talk between cancer and stroma cells allows for formation of a feed-forward loop, perpetuating cancer progression. Thus, the tumour microenvironment is an important factor in cancer development, and tumour stroma is another attractive target for PDAC treatment, potentially increasing the efficacy of chemotherapy. However, results from conducted studies are not clear cut. One of the first pieces of evidence of the potential benefits of targeting the stroma comes from nab-paclitaxel/gemcitabine studies, which showed a significant increase in the intracellular gemcitabine concentration due to decreased cancer-associated fibroblasts and stroma disruption facilitated by nab-paclitaxel [49]. As mentioned above, targeting multiple receptor tyrosine kinases, e.g., blocking of VEGFR and PDGFR with dovitinib, showed an improvement in therapeutic efficacy in mouse models, and clinical trials are currently ongoing [195,196]. Hedgehog pathway plays a pivotal role in cell survival and proliferation during development. Typically, it is repressed in mature pancreas; however, its activation has been observed during carcinogenesis. In addition, sonic hedgehog (SHH) and its downstream effectors take part in the formation of desmoplasia, contributing to decreased drug delivery [197,198]. Therefore, the Hedgehog pathway inhibition raised a lot of interest in terms of its potential to decrease the proliferation and invasion of PDAC cells [199]; however, its inhibition showed contradictory results. Very encouraging and promising results of the Hedgehog inhibition (via Smoothened) with an infinity pharmaceuticals inhibitor of sonic hedgehog (IPI-926) agent were obtained by Olive et al. [200], demonstrating a potent anti-tumour activity of the compound in a series of preclinical studies. Combined with gemcitabine or nab-paclitaxel, IPI-926 significantly increased drug delivery, reduced metastases and prolonged mice survival. Infinity pharmaceuticals conducted clinical trials of the compound in combination with gemcitabine and, despite the initials promising phase I/II results, the study needed to be discontinued due to decreased survival rate in the IPI-926/gemcitabine group compared to the gemcitabine alone group [201]. Interestingly, failure of Hedgehog targeting has been attributed to emerging evidence of the release of tumour restraining caused by the inhibition of this pathway. Currently, there are no FDA-approved Hedgehog inhibitors, nevertheless, clinical trials of chemotherapeutics and Hedgehog inhibitors are ongoing. Vismodegib (GDC-0049), an inhibitor of Hedgehog signalling pathway via inhibition of Smoothened, is under evaluation in combination with gemcitabine or gemcitabine and nab-paclitaxel for advanced and metastatic patients [202]. Its application as a sole agent has been also considered for neoadjuvant therapy [203]. Another molecule identified as possible target in the inhibition of cancer stroma is connective tissue growth factor (CTGF). Its overexpression in PDAC tissues has been confirmed, together with its ability to induce PSCs proliferation, migration and fibrogenesis mediated by chemokines activation [204]. SB225002, a Cxcr2 receptor inhibitor, prolonged survival of mice in in vivo studies [205]. Similarly, targeting the same receptor with a monoclonal antibody FG-3019 combined with gemcitabine showed a significant increase in gemcitabine efficiency in KPC mouse model [206], presenting a promising strategy for novel PDAC therapeutics. It is also known that pancreatic stellate cells (PSCs) and extracellular matrix (ECM) proteins actively participate in the formation of the tumour stroma [207] and in the activation of a plethora of cancer-promoting pathways leading to an increased tumorigenicity and chemoresistance by enhancing cancer stem-like phenotype [208,209]. Therefore, there are many strategies aiming to inhibit PSCs activation and ECM production. Among different agents, angiotensin II type 1 receptor blockers (ARBs) showed the most promising results. Candesartan, one of ARBs, was able to suppress PSCs activation as well as prolong patients’ survival for more than 6 months when combined with ACEIs (angiotensin I converting enzyme inhibitors) [210]. Another member of ARBs, losartan, apart from inhibiting PSCs activation, decreased levels of hyaluronan and collagen in the stroma, remodelling tumour microenvironment and increasing blood perfusion [211]. Matrix metalloproteinase inhibitors (e.g., marimastat) have also been tested, although no evidence of their superiority over gemcitabine has been provided [212]. Targeting of non-cellular stroma compartments, such as hyaluronic acid (HA), showed promising preliminary data. HA is a matrix component, which depletion might facilitate drug delivery by overcoming barriers caused by dense stroma. After promising results from a clinical trial of PEGPH20 (a PEGylated recombinant hyaluronidase which can deplete accumulated HA in tumours) and gemcitabine [213], PEGPH20 with Abraxane [214] combination is currently in progress. Overall, targeting the stroma and its particular components seems to be a promising and novel approach. Considering the significant contribution of dense tumour microenvironment in chemoresistance, agents aiming at releasing stroma may considerably improve tumour vasculature and drug delivery. However, there is some controversy regarding the safety of this strategy. Few studies have suggested that excessive relaxation of surrounding stroma may facilitate release of tumour cells, contributing to cancer dissemination [215]. Therefore, this aspect should be considered during design of pre-clinical and clinical studies.

## 7. Immunotherapy for Pancreatic Cancer

Another emerging option for treating advanced pancreatic cancer patients is immunotherapy. Induction of an anti-tumour immune response has been shown to be extremely effective in different advanced stage cancer types. However, immunotherapy trials in PDAC have shown conflicting results so far. An immunotherapy approach in pancreatic cancer therapy can be divided into a few categories: checkpoint inhibitors, vaccines, monoclonal antibodies, adoptive cell transfer, viruses, and use of cytokines. The first option, immune checkpoint inhibitors, by enhancement of stimulatory or blocking activity of immune system regulators, intensifies existing anti-cancer responses, enabling for better clearance of cancer cells. Programmed death receptor 1 (PD-1), a well as its ligand PD-L1, is one of the most important checkpoint pathways [224]. They are expressed on tumour-associated lymphocytes and are involved in suppression of immune responses observed during carcinogenesis, which is why they may be considered as one of the mechanisms of cancer immune resistance. Targeting this pathway should induce T cell activity and consequently cancer cell death. Therefore, antibodies targeting PD-1 receptor or PD-L1 are being investigated [225]. Phase I/II trials examining antibodies targeting another checkpoint inhibitor, cytotoxic T-lymphocyte-associated protein 4 (CTLA-4), (with e.g., ipilimumab, an FDA approved immunotherapy drug for melanoma) [226] are also ongoing. Nivolumab (anti-PD-1 antibody) alone (NCT02423954) or in combination with ipilimumab, anti-CTLA-4 Ab (NCT01928394); gemcitabine (NCT01473940), or other antibodies (NCT02526017, NCT02381314) are currently being tested. Studies of another anti PD-1 antibody, pembrolizumab (NCT02268825, NCT02305186) alone or in combination with gemcitabine and FAK inhibitor defactinib (NCT02546531), are also ongoing. Another combination of anti-PDL1 and anti-CTLA4 antibodies (durvalumab and tremelimumab respectively (NCT02558894, NCT02639026, NT02311361, NCT02527434) or durvalumab with mogamulizumab- anti-CCR4 Ab (NCT02301130) is being investigated in patients with advanced cancer, including pancreatic cancer. Unfortunately, preliminary data from the previously mentioned combinations indicate no significant improvement so far. To overcome the immunosuppressive activity of pancreatic cancer stroma, targeting CD40 has arisen as a novel strategy to increase anti-tumour activity. CD40 is a member of the tumour necrosis factor family expressed by immune cells, and its elevated expression and activity have been linked with different malignancies, including cancer [227]. Therefore, through immune system activation, targeting of CD40 can affect tumour growth. Several studies have been proposed, in which enhancing CD40 activity with its agonists may improve T-cell-dependent (macrophages activation and tumour stroma destruction) and independent immune responses and consequently induce cancer regression. A promising combination of gemcitabine and CD40 agonist antibody (CP-870,893) is being tested in clinical trials [228,229]. This combination enhances the accumulation of tumour-suppressive macrophages, increasing tumour regression. A Phase Ib/II trial of gemcitabine/nab-paclitaxel combined with indoximod (inhibitor of indoleamine-2,3-dioxygenase, tryptophan metabolite toxic to T cells) is ongoing, with preliminary data showing moderate and sustained activity [230]. Vaccine-based therapies are designed to enhance the immune system response against tumour-associated antigens. Unfortunately, no statistically significant clinical benefit over standard therapies has been achieved so far with vaccines, such as GV1001 [223] or PANVAC-V [231,232]. Nevertheless, a plethora of vaccine-based combinations clinical trials are currently ongoing (e.g., ipilimumab ±vaccine therapy [222], GVAX Pancreas vaccine (designed to secrete GM-CSF) ± nivolumab [233], GVAX, CRS-207 (vaccine targeting mesothelin protein) ± nivolumab (NCT02243371) or HyprAcute Pancreas (algenpantucel-L; NCT02405585) for both resectable and metastatic cancers. Various monoclonal antibodies are also currently used in cancer therapy. Cetuximab, an EGFR-targeting monoclonal antibody, despite promising phase I results, did not show any survival benefit in further studies [152]. Phase I and II clinical trials of, e.g., anti-HER3 antibody (MM-141, NCT02399137), Trop-2 antibody (IMMU-132, NCT01631552), or anti CA19-9 antibody (MVT-5873, NCT02672917), are currently being developed. Adoptive T cell therapy has emerged as another powerful tool in the enhancement of immune system responses. It is based on removal of the patient’s T cells, followed by boosting their activity through genetic/chemical re-engineering, and reintroduction into the patient. The modifications currently investigated include targeting anti-MAGE-A3 protein (NCT02111850), targeting NY-ESO-1 antigen (NCT01967823) or CAR T (chimeric antigen receptor T) cells reengineered to recognize mesothelin (NCT01583686). Virus therapies, such as ParvOryx (NCT02653313) or Reolysin, which replicates particularly in Ras-transformed cells (NCT02620423), are also currently assessed as anti-cancer tools, facilitating cancer cell self-destruction. Overall, different approaches to PDAC immunotherapy are presently being undertaken, with promising preclinical studies results. However, most of the studies are still in their early phases and much more effort needs to be made to fully assess their potential effectiveness and applicability in PDAC patients’ treatment.

## 8. miRNAs in PDAC Therapy

Recently, the developing field of miRNA investigation has attracted interest as another possibility for expanding the repertoire of PDAC treatments. It has been demonstrated, in several independent studies, that these short (18–22 nucleotide) non-coding RNAs can regulate expression and activation of multiple signalling pathways responsible for cell development, growth, differentiation and apoptosis, suggesting their possible involvement in carcinogenesis [234]. In fact, miRNA expression profiling showed abnormal expression of a plethora of different miRNA in several cancers including PDAC. Increased levels of pro-oncogenic as well as reduced expression of tumour suppressive miRNAs have been found in cancerous, as well as pre-cancerous pancreatic samples [235], suggesting their importance in PDAC development. Because each single miRNA targets multiple genes, causing alteration in their expression, targeting miRNAs provides encouraging approach for PDAC treatment, in which by targeting of one molecule, activation of multiple pathways may be altered. However, the same concept raises similar amount of concern, since alteration of that significant number of genes might cause severe adverse effects.

Several different expression profiles in pancreatic tissues from different sources (fresh frozen tissue, paraffin-embedded or fine-needle biopsy) showed a significant number of aberrantly expressed miRNAs compared to healthy pancreatic tissues [236,237]. Among many, increased expression of miR-21, miR-221, miR-155 and decreased levels of miR-146a, miR-34 and miR-145 were regularly detected across all the studies [238]. Moreover, overexpression of miR-155 and miR-21 has been correlated with advanced cancer stage and poor prognosis, with the latter being involved in the transformation from normal tissue to PDAC [239,240,241]. miR-155 has been found to be significantly upregulated in pancreatic tissues as well as in PanIN-2 and PanIN-3 samples [242]. Moreover, its expression correlated with PDAC patients’ survival and lymph node metastasis [243,244], suggesting the importance of miR-155 in PDAC carcinogenesis. By targeting the expression of molecules important in this process, such as suppressor of cytokine signalling 1 (SOCS1) or MLH1, miR-155 has been proposed as an important player in PDAC invasion and migration [244,245]. Its importance has been confirmed by in vitro studies, in which knockdown of miR-155 resulted in significant decrease in expression of EGFR and KRAS, proteins essential for PDAC development, as well as reduced cell proliferation and colony formation [246]. Similar correlation between cancer staging and miRNAs expression has been demonstrated for miR-221. Moreover, its expression has been also associated with metastasis and unresectable tumour status [247,248]. Inhibition of miR-221 significantly reduced PDAC cell proliferative capacity by targeting and blocking multiple genes, including *PTEN*, *P27* or *PUMA* [249]. Furthermore, increased miR-221 expression has been also detected in pancreatic stellate cells (PSCs) [250], suggesting its involvement not only in cancer cell proliferation but also in the tumour microenvironment. Similar observations have been reported for miR-146a; however, its expression has been found to be considerably decreased in PSCs [250]. It has been also shown that overexpression of miR-146a or its induction by isoflavone treatment, significantly decreased PDAC cell invasiveness by downregulation of, e.g., EGFR [251]. However, a separate study suggested that expression of miR-146a in PanINs was upregulated, suggesting its potential involvement in PDAC initiation [252]. All these findings make miR146a a controversial target for PDAC therapy. Three members of miR-34 family, miR-34a, miR-34b and miR-34c have been found downregulated in PDAC and were correlated with lymph node metastasis and poor survival [241,253]. Their impact on cancer cell proliferation, invasion, epithelial-mesenchymal transition (EMT) and cell cycle regulation through targeting molecules such as Notch or Bcl2 has been confirmed [254]. Interestingly, miR-34a downregulation can be partially attributed to epigenetic regulation (hypermethylation), suggesting demethylating agents as a possible therapeutic drugs [255]. In fact, isoflavone treatment resulted in miR-34a upregulation and consequently, induction of apoptosis and suppression of tumour growth [256]. A number of studies have considered miR-21 as a suitable target for PDAC therapy. Its elevated expression was found in 79% of pancreatic cancer samples, whereas only 8% of benign tumours expressed this miRNA [257]. Its activation triggers the response of multiple oncogenic signalling pathways, inducing cell proliferation, differentiation and exerting an anti-apoptotic role [258]. Similar to others, its expression has been correlated with PanIN progression, dismal prognosis, increased proliferation and invasion. Conversely, downregulation of the expression of miR-21 reduced proliferation of multiple cancer cell types [259] and it was shown to be beneficial in the adjuvant settings, increasing drugs activity. Importantly, gemcitabine resistance has been associated with miR-21 expression and thus this could be considered as a prognostic marker for gemcitabine response [257,260,261,262]. It has been demonstrated that co-delivery of gemcitabine and miR-21 silencers had a synergistic anti-tumour effect and presents a promising strategy for novel anticancer therapy. Taking into consideration the pivotal role of multiple miRNAs in a variety of carcinogenic processes, different approaches for the regulation of their activity have been considered. Nanoparticle delivery of tumour suppressing miRNAs, such as miR-150 or miR-34a resulted in reduction of cell proliferation and invasion, as well as was able to suppress tumour growth [263,264]. Analogously, combination of miR-21 and miR-221 antisense nucleotides reduced growth of primary tumours and significantly inhibited metastasis [265]. Recently, co-delivery of miRNAs and chemotherapeutics emerged as another promising strategy. In particular, co-administration of miRNAs (or their inhibitors) involved in chemoresistance seems an attractive approach. Co-delivery of miR-205 and gemcitabine was able to reverse this resistance and reduce proliferation and invasion of highly resistant PDAC cell lines such as MiaPaCa-2 or Capan-1 [266]. Similarly, targeting of miR-21 using lentiviral vectors stimulated angiogenesis, enhanced gemcitabine delivery and provoked tumour regression [259,260,261]. All of these results make miR-21 a promising target, which needs to be further evaluated in more advanced clinical studies. Another strategy in targeting miRNAs for anti-cancer therapy is the use of natural agents [267], such as the aforementioned isoflavone, curcumin or 3,3′-diindolylmethane (DIM). It has been formerly demonstrated that isoflavones possess anti-cancer activity. Considering our current knowledge, miRNAs alteration may be one of the mechanisms responsible for this activity. As previously mentioned, treatment with the isoflavone genistein was able to suppress tumour growth through upregulation of miR-34a expression. Similarly, elevated expression of miR-146a and miR-200, as well as a decrease in miR-27a levels were detected after isoflavones treatment, which resulted in reduction of cell proliferation and invasion and increased sensitization of cells to gemcitabine treatment [251,268]. Anti-cancer activity of DIM is also exerted via regulation of different miRNAs, including miR-200, miR-221 or miR-146a. A decrease in cell proliferation and migration, reversal of EMT and sensitization to gemcitabine were induced after exposure of cancer cells to DIM [251,269]. Similar effects could be achieved with the curcumin analogue difluorinated-curcumin (CDF), which increased curcumin bioavailability. CDF treatment elevated expression of miR-101, miR-146a and miR-200 and decreased miR-211 levels. This activity results in inhibition of pancreatic cancer cell growth and migration, decreased colony formation, as well as downregulation of a plethora of pathways pivotal for PDAC progression, including EGFR, ERK or KRAS expression [270,271]. Other natural agents, such as Brucein D, resveratrol or rosemary extracts exerted similar effects on pancreatic cancer cells, through regulation of different miRNAs [272,273]. Overall, targeting miRNAs either by their re-expression or inhibition seems a novel and promising strategy in pancreatic cancer treatment. Furthermore, this approach has been shown to enhance cancer cell response to chemotherapy, by reducing cancer chemoresistance. However, there is a need for in-depth preclinical and clinical studies to assess the efficiency and safety of this strategy. Alteration of single miRNA can result in the cascade of changes in activity of downstream effectors, contributing to elevated adverse effects. Moreover, considering the correlation between the expression of most of miRNAs, PDAC stage and patients’ OS, miRNAs levels should be also further explored as novel, predictive biomarkers.

## 9. Second-Line Therapies

Limited options are available for patients whose disease has progressed after gemcitabine-based first line treatment. Oxaliplatin-based therapies are usually offered in these cases, but good performance status is a critical factor. The beneficial effect of oxaliplatin in addition to 5-FU and folinic acid, over individual therapies, has been observed in several trials (CONKO-01, CONKO-03); with an acceptable safety profile and almost doubling of the survival period [274,275]. However, contradictory results were obtained in the PANCREOX study, in which addition of oxaliplatin to mFOLFOX6 (infusional FU/LV) showed no benefit in patients who progressed on gemcitabine-based first line therapy [276]. A single-arm phase II study of docetaxel and oxaliplatin (DocOx) in gemcitabine-refractory patients has recently been conducted, and a median overall survival time of 10 months was noted [277]. Second line combinations of capecitabine and oxaliplatin (CapOx) have been also considered, with encouraging activity and safety profile of the combination [278]. FOLFOX treatment (Leucovorin, 5-FU and Oxaliplatin) also proved to be an efficient (mOS of 4.3 months) and considerably safe second-line treatment for metastatic patients with good PS [279]. Its activity was comparable with yet another agent tested for second-line PDAC treatment, FOLFIRI (Leucovorin, 5-FU and Irinotecan), which increased survival by approximately six months [280,281]. A combination of capecitabine and JAK-1 and JAK-2 inhibitor ruxolitinib, administered to patients who already received gemcitabine, is being investigated in a phase III JANUS study [184]. Studies analysing second-line therapies after FOLFIRINOX failure are also under investigation. Gemcitabine and nab-paclitaxel, as well as maintenance capecitabine, have shown promising results and further studies are planned [282,283,284]. An alternative option for a second line treatment was proposed in the NAPOLI-1 trial [285], in which nanolioposomal irinotecan combined with 5-FU and folinic acid significantly increased OS and PFS in a phase II study. Taken together, no optimal second-line therapy has been determined. Therefore, there is an increasing interest in defining most favourable strategy for treatment of advanced PDAC patients who failed to respond to conventional therapies.

## 10. Conclusions

Despite efforts made to develop more effective therapeutic strategies for PDAC, it still remains one of the most fatal malignancies, for which incidence constantly rises. Even though advances have been made in screening and treatment of other cancer types, PDAC therapy has not experienced significant improvement in the last decades. Gemcitabine and its doublets failed to provide considerable survival benefit. Multidrug therapies—Abraxane and FOLFIRINOX—have been recently developed moderately improving patients’ outcomes; however, their efficacy still remains low and their usage is coupled with elevated adverse effects. Therefore, there is an urgent need for the development of novel and more effective treatments.

Thus far, tumour resection supported by adjuvant therapy has presented the only curative option for PDAC patients. However, less than 20% of patients have resectable tumours at the time of diagnosis, caused by local and distant metastasis. Therefore, efforts are being made to increase the percentage of patients able to undergo this procedure. Very early dissemination of pancreatic cancer provides ground for the applicability of neoadjuvant therapies, potentially increasing resection rates. However, despite theoretical advantage, no straightforward evidence of clinical applicability of neoadjuvant therapies is available. Preoperative treatment demonstrated considerable benefit in the increase in R0 resections, the main survival predictive factor. Unfortunately, the significant increase in R0 resection rates in patients subjected to neoadjuvant therapies did not fully translate into patients’ survival benefits. The lack of consensus on effectiveness in resectable patients, as well as contradictory results for BRPC, makes this strategy highly debatable. The controversy of the feasibility of neoadjuvant therapies is due to the inconsistency of the design of clinical trials and difficulty in data interpretation. Lack of standardization and perioperative quality control makes it difficult to properly assess the applicability of neoadjuvant treatments. Inconsistency in accurate tumour classification, varying between centres, single arm phase I/II trials, limited sample size and mostly retrospective data, analysing patients with different disease context, impairs proper data comparison, resulting in lack of consent on the use of neoadjuvant regimen for PDAC patients. In addition, currently available imaging tools do not accurately assess tumour burdens and make it difficult to distinguish treatment-induced fibrosis from extended tumour, disabling proper distinction between down-staged and untreated cancer and potentially depriving part of the population from successful R0 resections. Additionally, most of the studies involved the use of one or two therapeutics; however, recent evidence suggests that multidrug treatments (i.e., PEFG, PDXG or FOLFIRI) yielded significantly higher response rates, showing superiority in various retrospective studies. Therefore, prospective complex evaluation of multi-agent strategy should be more widely explored. Overall, it has been suggested that if properly designed, neoadjuvant treatment followed by surgery may increase five-year survival rates up to 40%. Therefore, the standardization of staging procedures and the initiation of higher number of prospective phase III trials might significantly add to patients’ survival. Based on available data, several studies are focusing on providing an algorithm of action improving the decision-making and consequently providing better outcomes; however, so far only marginal benefit has been demonstrated [286]. An Alliance trial is currently being evaluated in order to assess the effectiveness of modified FOLFIRINOX as neoadjuvant agent and to establish reproducible standards for BRPC therapy. In general, despite the controversy and reluctance of some centres to apply neoadjuvant therapy to PDAC patients, this approach is supported by the National Comprehensive Cancer Network (NCCN) in the United States [84].

The grim prognosis for PDAC patients and the disappointing therapeutic results are attributed to the highly proliferative and chemoresistant nature of PDAC. Therefore, targeting signalling pathways and mechanisms dysregulated during PDAC development has emerged as a new possibility and has opened the door for more personalized treatments. In the last years, the strategy of combining targeted agents with chemotherapy has been widely explored; however, although successfully introduced in multiple solid cancer types, targeted therapy failed to demonstrate any clinical benefit for pancreatic cancer patients. The only exception, erlotinib (Traceva), although only moderately improving OS, provided bases for further exploration of therapeutic possibilities. Huge heterogeneity and complexity of PDAC is regarded to be a major clinical obstacle in the development of successful therapies. Targeting individual molecules is not a sufficient approach, as it is counteracted by upregulation of members of adjacent pathways, contributing to therapy failure. Therefore, strategies combining chemotherapy with targeting multiple targets could considerably diminish this drawback. However, unpredictable adverse events of such a broad interference should not be neglected. Hitherto, most of the conducted studies were designed based on gemcitabine activity. Considering that gemcitabine is no longer the drug of reference, the focus of future studies should be placed on targeted therapies involving Abraxane or FOLFIRINOX, potentially improving achieved outcomes. In PDAC, high mutational variability is observed not only between patients, but also throughout individual samples. Therefore, another major flaw of current clinical trials is the lack of patients’ selection and classification into prognostic subpopulations. In fact, less than 10% of conducted studies selected their patients on the basis of predictive molecular markers [287]. The individualised molecular pancreatic cancer therapy (IMPACT) trial is currently being evaluated in order to stratify patients and allow for more personalized treatments [288]. Additionally, a recent meta-analysis has shown that only a small subset of trials (40%) have been conducted after confirming drug efficacy in thorough pre-clinical studies. A small percentage of studies (30%) formulated their hypothesis based on in vivo studies, whereas the vast majority was based on in vitro, cell line studies [289]. Therefore, although most of the studies demonstrated promising results during preclinical evaluation, the vast majority failed to proceed to more advanced clinical studies due to the lack of efficiency. Therefore, better models should be developed to more accurately recapitulate human disease and make pre-clinical studies more relevant.

On the other hand, novel, potent therapeutic targets should be explored. Considering the high variety of miRNAs aberrantly expressed in PDAC and their role in the control of cell proliferation, invasion and apoptosis, the strategy of altering their expression and activity in order to prevent cancer development and progression seems promising. Synthetic nanoparticle delivery of miRNAs, which are downregulated in cancer tissues, as well as inhibition of overexpressed miRNA, mainly with the use of natural agents has been explored. Both approaches showed promising in vitro and in vivo results; however, we are currently lacking knowledge about possible adverse events. Considering that each miRNA has multiple targets, their alteration might cause unpredictable modifications in many pathways, contributing to fatal consequences. Therefore, more advanced pre-clinical and clinical studies are needed to fully elucidate the potential of miRNAs modulation in PDAC therapy. Boosted research and clinical studies should be also focused on the role of pancreatic cancer stem-like cells, a subpopulation of slow-cycling highly metastatic cells showing increased chemoresistance. The ability to control this subpopulation of cancer cells, responsible for enhanced aggressiveness and invasion potential, could be of great clinical value. If successful, novel strategies targeting this subpopulation would make a breakthrough in PDAC therapy.

Altogether, pancreatic cancer is a complex disease that should be managed with an integrative approach. In order to fulfil the goal set by clinicians and scientists to double PDAC patients’ survival by 2020, multidisciplinary strategy, determining best palliative techniques and tailoring specific therapeutic strategies aimed at specific subpopulations of patients is of crucial importance. Close collaboration between oncologists, radiologists and surgeons would allow for accurate patients’ classification into proper modality. Disease stage, but also mutations, performance and nutrition status should also be considered.

## Figures and Tables

**Table 1:** Gemcitabine-based combination therapies.

Treatment	Phase	*n*	OS (Months)/Response Rate (%)	Outcome	*p*	Reference
Gem vs. 5-FU	R FL III	126	5.65 vs. 4.4	FDA approved	0.0025	[22]
Gem-5FU vs. gem	FL III	322	6.7 vs. 5.6	No statistically significant improvement in OS	0.09	[65]
FOLFIRINOX	R II/III	342	11.1 vs. 6.8	FDA approved	<0.001	[60]
Abraxane	R III	861	8.5 vs. 6.7	FDA approved	<0.001	[49]
Erlotinib + gem/gem	R III	569	6.2 vs. 5.9	FDA approved	0.038	[42]
Gem + cisplatin/gem	R III	195	7.5 vs. 6.0	Improved survival, but not statistically significant	0.15	[33]
	R III	400	7.2 vs. 8.3	Failed to demonstrate improvement	0.38	[32]
PEFG vs. gem	III	99	38.5% vs. 8.5%	Little sample size	0.0008	[37]
Gem + oxaliplatin	III	313	9.0 vs. 7.1	Significant improvement in response rate and PFS, but not statistically significant OS	0.13	[30]
Gem + capecitabine vs. gem	III	319	8.4 vs. 7.2	Not statistically significant improvement in OS	0.234	[44]
	III	533	7.1 vs. 6.2	Alternative treatment for patients with good PS	0.08	[34]
S-1 + gem/gem	III	834	9.7 vs. 8.8	Not inferior to gemcitabine. Approved in Japan as alternative	<0.001	[39]
Gem + irinotecan	III	360	6.3 vs. 6.6	Good tumour response but no improvement in OS	0.789	[29]

FDA, Food and Drug Administration; R, randomized; PS, performance status; OS, overall survival; PFS, progression-free survival; gem, gemcitabine; PEFG, cisplatin, epirubicin, fluorouracil, and gemcitabine combination.

**Table 2:** Selected targeted therapies and immunotherapies for PDAC.

Drug Target	Treatment	Phase	*n*	OS	Comment	*p*	Reference
KRas pathway inhibitors							
KRAS (farnesyl transferase)	Tipifarnib + gem vs. gem	R III	688	193 vs. 182 (days)	Acceptable toxicity profile, but no statistically significant differences in survival parameters	0.75	[163]
MAPK	Selumetinib + erlotinib 2nd line	SA II	46	7.5	Modest antitumor activity. Specific molecular subtypes may provide greatest benefit	–	[216]
MAPK	Trametinib + gem vs. gem	R II	160	8.4 vs. 6.7	No statistical difference in OS, PFS and response rate was observed	0.453	[165]
MAPK	Selumetinib + cape vs. cape 2nd line	R II	70	5.4 vs. 5.0	No improvement in OS	0.92	[164]
MAPK	Sorafenib + gem vs. gem		104	9.2 vs. 8.0	No statistical significance was achieved in all parameters studied	0.231	[217]
mTOR	Everolimus + erlotinib	SA II	16	2.9	Disease progression observed in 15 patients. Study stopped due to impossibility to reach preplanned OS of 6 months	–	[173]
PI3K	Rigosertib + gem vs. gem	R II/III	160	6.1 vs. 6.4	Study was discontinued due to no significant difference in survival	NR	[168]
Growth factor receptors inhibitors							
EGFR	Erlotinib + gem vs. gem	R III	569	6.2 vs. 5.9	FDA approved	0.038	[42]
EGFR	Cetixumab + gem vs. gem		743	6.3 vs. 5.9	Combination arm did not achieve significance in improvement of OS	0.19	[152]
EGFR/IGFR	Cixutumumab + erlotinib + gem vs. erlotinib + gem	R Ib/II	116	7.0 vs. 6.7	Dual inhibition of EGFR and IGFR did not improve OS or PFS	0.64	[161]
EGFR	Gefitinib + gem	SA II	53	7.3	Promising results, especially in patients with PTEN expression.	–	[153]
HER-2	Trastuzumab + cape	SA II	17	6.9	No improvement in mOS or PFS; low number of patients and HER2 expression	NR	[155]
TK	Dasatinib	SA II	51	4.7	No activity of single agent dosatinib in metastatic PDAC, no improvement in OS and PFS	–	[216]
TK	Lapatinib + gem	SA II	29	4	No improvement in survival, small case sample	–	[156]
IGFR	Ganitumab + gem vs. gem	R III	800	7.0 vs. 7.2	No improvement in all assessed parameters	0.494	[162]
Angiogenesis inhibitors							
VEGFR	Axitinib + gem vs. gem	R III	632	8.5 vs. 8.3	No significant survival benefit compared to single agent gem	0.544	[176]
VEGF-A	Bevacizumab + gem + erlotinib vs. gem + erlotinib	R III	301	7.1 vs. 6.0	Despite improvement in PFS could be observed (*p* = 0.0002), no statistically significant difference in OS was achieved	0.209	[218]
VEGF	Aflibercept + gem vs. gem	R III	587	6.5 vs. 7.8	Discontinued due to no improvement in primary end point, OS	0.159	[180]
Inhibition of tumour stroma							
Matrix metalloproteinase	Matrimastat + gem vs. gem	R III	239	5.4 vs. 5.4	No significant differences in all assessed parameters	0.95	[212]
SHH	Vismodegib + gem vs. gem	R Ib/II	106	6.9 vs. 6.1	No difference in PFS, OS or response rate was noted	0.84	[202]
PSCs	Candesartan + gem	SA II	35	9.1	Treatment was well tolerated but failed to show significant activity	–	[219]
Hedgehog (Smoothened)	IPI-926 + gem vs. gem	R Ib/II	122	–	Decrease in survival in IPI-926 arm caused closure of study	NR	[220]
Hyaluronic acid	PEGPH20 + gem	Ib	28	6.6	Well tolerated, may be beneficial, especially for patients with high HA levels (13 months OS)	–	[213]
	PEGPH20/Abraxane vs. Abraxane	R II	237		Ongoing		[214]
		R III	420		Ongoing		
Other targets							
JAK/STAT	Ruxolitinib + cape vs. cape	R II	127	4.5 vs. 4.2	Well tolerated, slight, but significant improvement in OS and PS	0.011	[183]
	2nd line therapy	R III	270		Phase III on larger population is ongoing		[184,221]
γ-secretase	RO4929097 2nd line	SA II	18	4.1	Study was discontinued as the primary endpoint-survival rate at 6 months—was not promising (27.8%)	–	[190]
Immunotherapy							
CTLA-4	Ipilimumab + GVAX vaccine vs. ipilimumab	R Ib/II	30	5.7 vs. 3.6	Despite the enhancement of the T cell repertoire (*p* = 0.031), no significant increase in OS or PFS was noted	0.51	[222]
Telomerase vaccination	GV1001 + gem + cape/gem + cape	R III	1062	8.4 vs. 6.9	No significant improvement in OS has been achieved	0.11	[223]

SA, single arm; R, randomized; OS, overall survival; PFS, progression-free survival; RR, response rate; cape, capecitabine; gem, gemcitabine.

## Abbreviations

ARBs	Angiotensin II receptor blockers
BRPC	Borderline resectable pancreatic cancer
CA19-9	Carbohydrate antigen 19-9
CAR T	Chimeric antigen receptor T
CXC	Chemokine
CDF	Difluorinated-curcumin
CDKN	Cyclin-dependent kinase inhibitor
CRP	C-reactive protein
CSF1R	Colony stimulating factor 1 receptor
CT	Computed tomography;
CTGF	Connective tissue growth factor
CTLA4	Cytotoxic T-lymphocyte-associated protein 4
DIM	3,3′-diindolylmethane
DDL4	Delta like canonical notch ligand 4
ECE1	Endothelin converting enzyme 1
ECF	Extracellular matrix
EGF	Epidermal growth factor
FAK	Focal adhesion kinase
FDA	Food and Drug Administration
FLT3	Tyrosine-protein kinase
Gy	Gray
HA	Hyaluronic acid
hENT	Human equilibrative nucleoside transporter
IGF1R	Insulin-like growth factor 1 receptor
JAK	Janus kinase
LAPC	Locally advanced pancreatic cancer
LN	Lymph-node ratio
LV	Leucovorin
MAGE-A3	Melanoma-associated antigen 3
MAPK	Mitogen-activated protein kinase
MLH1	MutL homolog 1
neoCRT	Neoadjuvant chemoradiotherapy
OS	Overall survival
PanIN	Pancreatic intraepithelial neoplasia
PARP	Poly ADP ribose polymerase
PD	Pancreaticoduodenectomy
PDAC	Pancreatic ductal adenocarcinoma
PDGFR	Platelet-derived growth factor receptor
PD-L1	Programmed death-ligand 1
PFS	Progression free survival
PS	Performance status
PSCs	Pancreatic stellate cells
PV	Portal vein
RT	Radiotherapy
SMAD4	Mothers against decapentaplegic homolog 4
SMV	Superior mesenteric vein
SOCS1	Suppressor of cytokine signalling 1
PUMA	p53 upregulated modulator of apoptosis
TK	Tyrosine kinase
TNM	Tumour node metastasis
VEGFR	Vascular endothelial growth factor receptor
